# High spatial resolution mapping of individual and collective localized surface plasmon resonance modes of silver nanoparticle aggregates: correlation to optical measurements

**DOI:** 10.1186/s11671-015-1024-y

**Published:** 2015-08-04

**Authors:** Carlos Diaz-Egea, Rafael Abargues, Juan P. Martínez-Pastor, Wilfried Sigle, Peter A. van Aken, Sergio I. Molina

**Affiliations:** Instituto de Microscopía Electrónica y Materiales, Departamento de Ciencia de los Materiales e I. M. y Q. I., Facultad de Ciencias, Universidad de Cádiz, Campus Río San Pedro, s/n, 11510 Puerto Real, Cádiz Spain; UMDO (Unidad Asociada al CSIC-IMM), Instituto de Ciencia de los Materiales, Universidad de Valencia, PO Box 22085, 46071 Valencia, Spain; Max Planck Institute for Intelligent Systems, Stuttgart Centre for Electron Microscopy, Heisenbergstraße 3, 70569 Stuttgart, Germany

**Keywords:** Plasmonics, Surface plasmon resonance, Silver nanoparticles, EFTEM, Nanoparticle clusters, Plasmonic modes, 78.67.Bf, 61.46.Df, 87.64.Ee

## Abstract

Non-isolated nanoparticles show a plasmonic response that is governed by the localized surface plasmon resonance (LSPR) collective modes created by the nanoparticle aggregates. The individual and collective LSPR modes of silver nanoparticle aggregated by covalent binding by means of bifunctional molecular linkers are described in this study. Individual contributions to the collective modes are investigated at nanometer scale by means of energy-filtering transmission electron microscopy and compared to ultraviolet–visible spectroscopy. It is found that the aspect ratio and the shape of the clusters are the two main contributors to the low-energy collective modes.

## Background

The optical properties of the noble metal nanoparticles (NPs) are dominated by localized surface plasmon resonances (LSPR) [1]. The LSPR frequency of gold and silver NPs are typically in the near UV, visible or infrared part of the spectrum, but the exact value of this frequency depends on several factors: on the one hand, on the dielectric constant of the surrounding media, and on the other hand, on material, size, shape, and aspect ratio of the NPs [2]. If the NPs are close to each other, even creating clusters, then the LSPR is also controlled by the distance between NPs and the aspect ratio and shape of the NPs clusters [3, 4].

Two main modes can be identified for clusters of NPs when they are not touching each other: a bonding mode that appears in the area between particles and depends on the interparticle distance [4, 5] and an antibonding mode that can be sensed in the extremes of the long axis of the cluster and depends on the aspect ratio of the group [6]. In this work, silver NP aggregates have been created. They were analyzed at a large scale by UV–vis spectroscopy (UV–vis) and at nanometer scale by energy-filtering transmission electron microscopy (EFTEM). That allowed the identification of the plasmonic modes that contribute to the global optical behavior of the system. The analysis of clusters with increasing number of NPs showed that indeed the aspect ratio of the clusters determines the collective mode. The importance of the shape of the cluster was also studied and discussed.

## Methods

For the work presented here, four samples were prepared containing Ag NPs that were either mainly isolated or clustered in different group sizes. The fabrication process is based on the covalent binding among NPs by means of bifunctional molecular linkers. OH-terminated Ag NPs were synthesized as building blocks according to reference [7]. Briefly, 20 mg of PVA is suspended by ultrasonication in a solution of 1 mg of AgNO_3_ in 10 mL of methanol. The resulting solution is then exposed to MW at 300 W for 40 s in a closed vial and 20 ml of water is added. Here, PVA works also as capping agent. For the formation of aggregates, oxalic acid was used as the linker to form clusters of NPs with an average interparticle spacing of 0.9 nm. Typically, 40 mL of as-prepared silver nanoparticles was reacted with 4 mL of 0.1 mM freshly prepared oxalic acid aqueous solution in a thermostatic bath at 40 °C. The size of cluster was controlled with the reaction time. The first sample was taken at the beginning of this process, when most NPs are isolated. Sample# 2 was obtained after a reaction time shorter than 3 h to make sure that most clusters did not get bigger than just two or three NPs. Sample# 3 was taken after longer reaction time for bigger clusters to be formed. After 40 h, the reaction was considered to be complete obtaining big groups of Ag NPs (sample# 4). The fabrication method is thoroughly described in a previously presented work [8].

In order to perform plasmonic analysis at a nanometric scale, one can perform scanning transmission electron microscopy (STEM) in combination with electron energy loss spectroscopy (EELS) and EFTEM. Both techniques have advantages and disadvantages, the main difference is that EELS has better energy resolution and can sense weaker signals than EFTEM, but EFTEM can directly perform high spatial resolution analysis of larger areas with reasonable energy resolution [9, 10]. For the work presented here, the EFTEM images were acquired in the Zeiss SESAM microscope [11, 12] (Oberkochen, Germany) using the same conditions as described in ref [10]. Both monochromator resolution and energy filter slit widths were set to 0.2 eV. An energy-filtered image was acquired at every 0.2 eV starting from 4 eV down to −0.6 eV, in order to minimize the afterglow effects of the scintillator/CCD camera system. The specimen drift between individual images was corrected by application of a script described in ref [13].

## Results and discussion

**Fig. 1:**
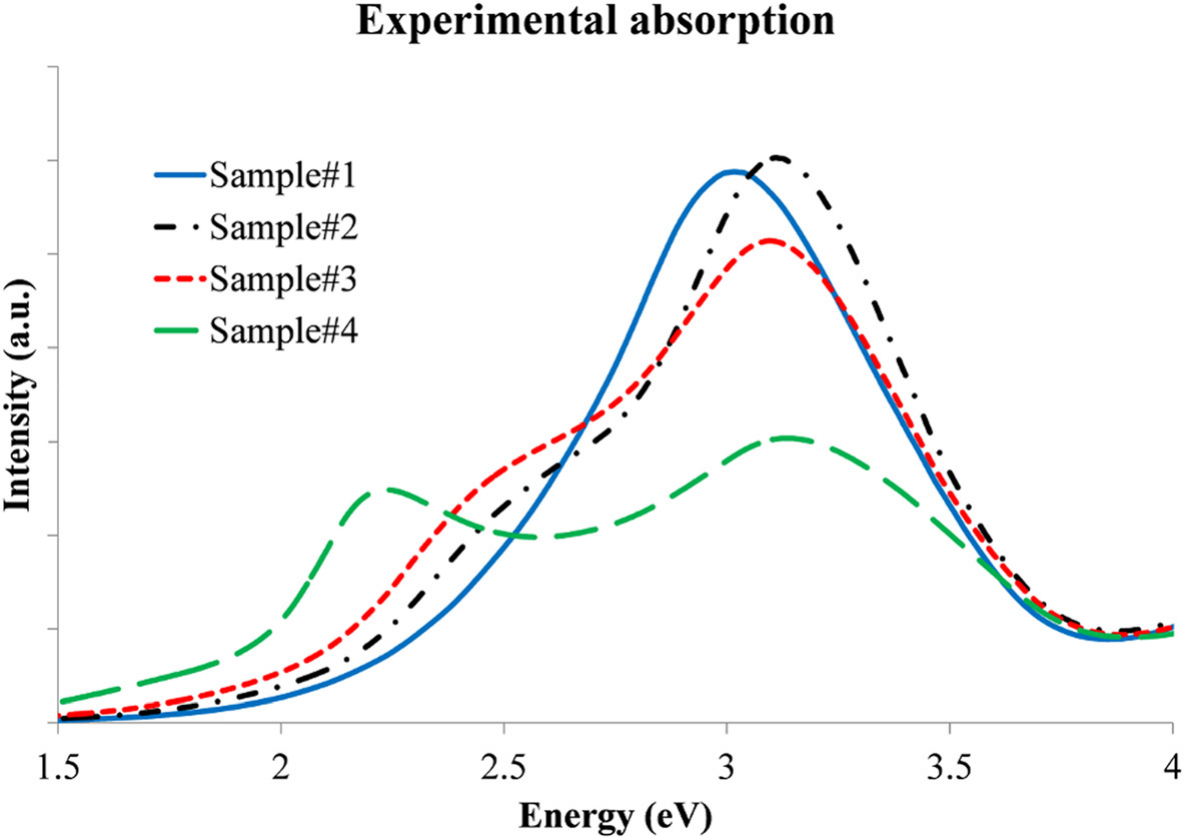
UV–vis absorption spectra of Ag nanosphere clusters. The cluster sizes range from almost only individual particles (sample# 1) to bigger number of NPs (sample# 2, sample# 3, and sample# 4)

The optical response of the different samples was measured by UV–vis spectroscopy (Fig. 1). The results are presented with respect to energy values instead of wavelength to make the comparison with EELS more direct. Sample# 1 is the one with predominantly single Ag NPs. The UV–vis absorption spectrum shows a single LSPR band at 3.1 eV. This value is somewhat lower than expected for a spherical Ag NP in air. A possible explanation comes from the fact that in this sample, there are a large number of slightly elongated NPs which would shift the global LSPR response to lower energy values [14–16]; in fact, the LSPR band is slightly asymmetric on its low-energy side. Sample# 2 and sample# 3 show similar results with a clear shoulder appearing at around 2.5 eV preceding the 3.2 eV peak getting weaker for sample# 3. The shoulder is due to a collective mode expected after the formation of small clusters of NPs. Three main contributions to this collective mode can be proposed: when NPs are not touching but very close, they couple creating a new low-energy mode [4]. The energy value of this new mode depends on the spacing between NPs decreasing as the NPs get closer to each other [17, 18]. The second and third possible contributing effects to the collective mode originate from the way the clusters of NPs are formed. The aspect ratio of a cluster determines the response of the entire group with lower energies for larger ratio values [6]. Finally, also the shape of the cluster controls the LSPR of the assembly. In the same way as a triangular NP shows lower energy modes than a sphere [19], a triangular or rhomboidal cluster has lower energy modes than a spherical one [20]. The aspect ratio of the cluster seems to be the dominant contribution to the collective mode in sample# 4 and that is why the low-energy mode of this sample is as low as 2.25 eV. These results are in line with previously presented data [8], even if that work was focused on the inter-NP distance and not on the cluster shape.

**Fig. 2:**
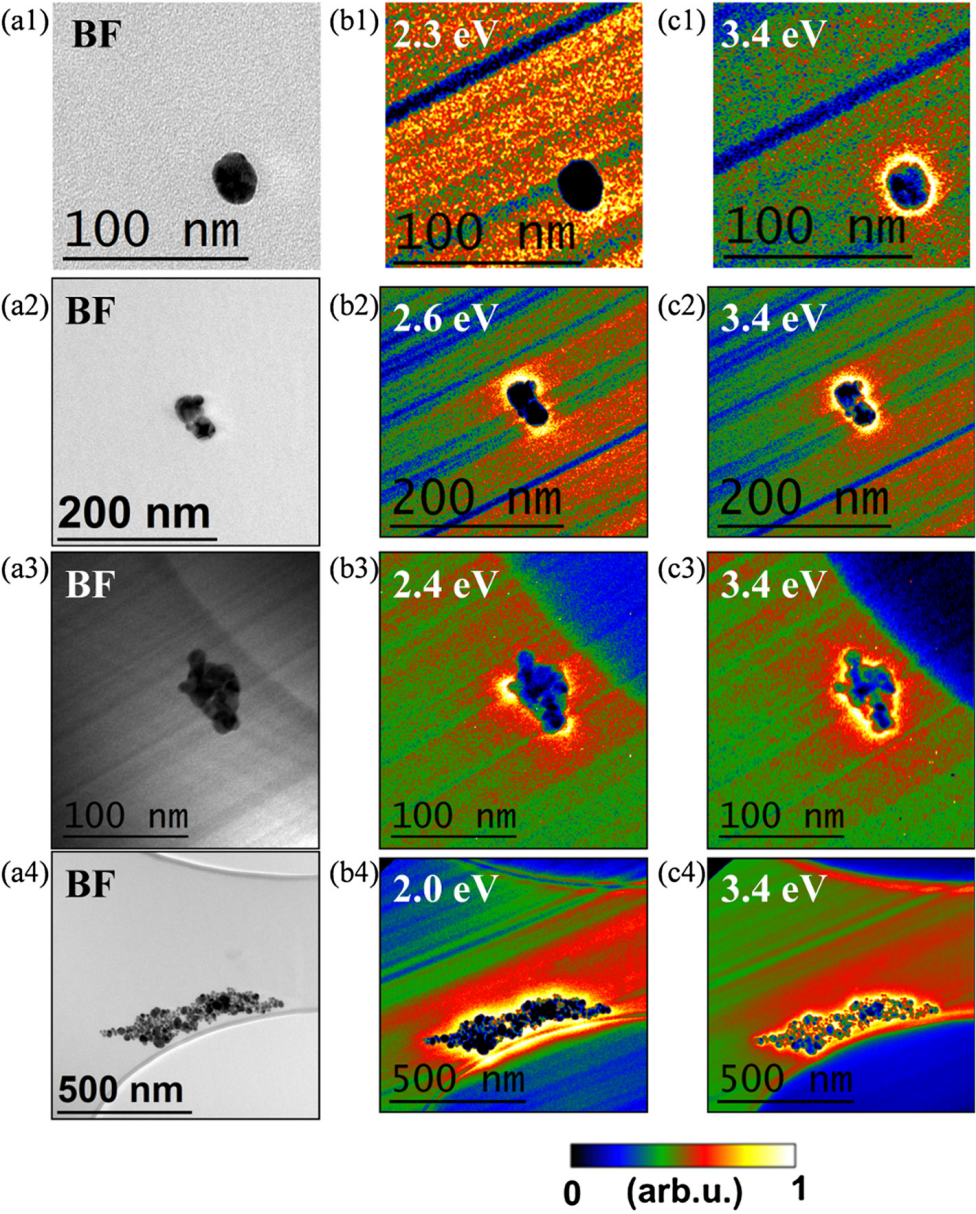
**a** Bright field transmission electron micrograph and **b**, **c** EFTEM images of the (1) isolated Ag NP, (2) small cluster, (3) medium size cluster, and (4) large cluster. *Inclined stripes* are artifacts originating from scattering at the monochromator slit. The *color bar* applies to all of the EFTEM images

A comprehensive study of the plasmonic modes of the Ag NP aggregates was done at a nanometer scale by EFTEM. Figure 2 summarizes the results obtained. They have been numbered 1, 2, 3, and 4 corresponding to samples 1 to 4, respectively. For each of them, three images labeled a, b, and c show the bright field (BF) TEM image and two EFTEM images.

Case number 1 shows a pseudo-spherical NP of approximately 25 nm diameter as can be seen in the BF TEM image shown at a1. b1 shows the energy-filtered map created at 2.3 eV where it can be seen that there is no resonance around the NP as it was expected for a standing alone spherical NP. Another energy-filtered map was acquired at 3.4 eV; the result is a glow around the NP which corresponds to the well-known dipolar mode for silver nanospheres in air [21].

The second case is a pair of nanospheres that are very close together. In fact, there are another two small NPs attached to the dimer, but they have very little influence on the response of the group. a2 shows the BF TEM image of the cluster; two NPs can be seen with a spherical shape of approximately 30 nm of diameter, and there are also two small NPs of 13 nm of diameter attached to them. The first EFTEM image shown in b2 reveals the presence of a strong field concentration on the extremes of the long axis of the dimer at 2.6 eV. As it was done in the first case, another energy-filtered map was taken at 3.4 eV; it shows the expected glow around the group.

The third case is a medium-sized cluster with some 20 NPs between 10 and 20 nm in diameter. The cluster has a rhomboid shape measuring around 40 and 70 nm along its short and long axes (see the BF TEM image in Fig. 2 a3). It can be seen that the EFTEM image at 2.4 eV exhibits bright areas at the sharp corners of the cluster (Fig. 2 b3) in contrast to a homogenous distribution in the EFTEM image taken at 3.4 eV (Fig. 2 c3).

The last selected case is a big cluster of 200 NPs approximately ranging from 6 to 50 nm in diameter. The size of the cluster is around 750 by 100 nm along its long and short axes, respectively (Fig. 2 a4). The EFTEM maps at 2.0 (Fig. 2 b4) and 3.4 eV (Fig. 2 c4) both exhibit a strong electric field concentration not only around the cluster perimeter but also inside the cluster in the case of the image at 3.4 eV

The most evident observation from the EFTEM images is that there is a glow around every NP or NP group for all four samples at 3.4 eV. This was observed in the series of images c1, c2, c3, and c4 in Fig. 2. We attribute this to the well-known dipolar mode of single silver NPs [20–22]. In the case of sample 1, this is the only visible contribution. Two other observations can be made by inspecting the series of images from b1 to b4: no resonance is observed at energies below 3.4 eV around single nanospheres while it appears in NP groups with a characteristic energy decreasing with increasing the size and aspect ratio of the cluster. It is interesting to analyze the diversity of contributions to the collective mode. In the case of the pair of NPs, Fig. 2 b2 shows that the resonance at 2.6 eV takes place predominantly at the extremes of the long axis of the dimer suggesting that it corresponds to a longitudinal collective mode. Something different is observed in Fig. 2 b3, where the rhomboidal shape of the cluster seems to create collective azimuthal and polar plasmon modes at the corners of the cluster around 2.4 eV. This is similar to what happens with single standing nanodecahedra [23] or more simply with triangles [19, 24]. The cluster analyzed at b4 is so large and complex, with some 200 NPs of all sizes, that it is difficult to conjecture the origins of the collective mode at 2.0 eV.

**Fig. 3:**
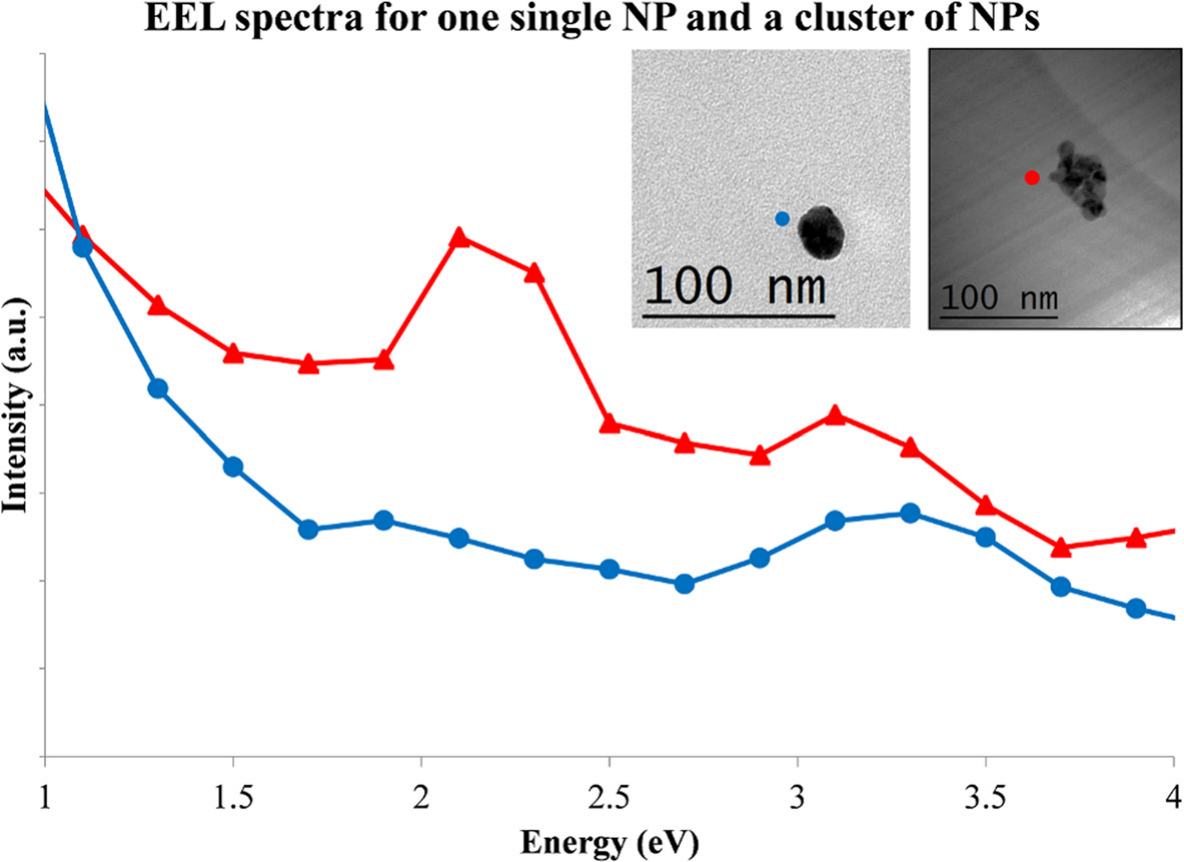
Electron energy-loss spectra extracted from the EFTEM series. The *blue curve* shows the result in the vicinity of an isolated NP; the exact location is marked with a *blue dot* in the bright field image shown in the inset. The *red curve* shows the result nearby a corner of a cluster of NPs, and the *red dot* signals the exact location

A more graphical interpretation of the results is shown in Fig. 3. The blue line displays one EEL spectrum extracted from the EFTEM series previously shown for sample# 1. The location of the spectrum was selected nearby the surface of the NP to show the dipolar plasmonic mode clearly located at 3.4 eV. There is a small shoulder around 2.0 eV that could be explained by the fact that the NP is not perfectly spherical. The red line is an EEL spectrum extracted from the EFTEM data of sample# 3. The location is at one of the corners of the cluster. Two modes coexist at this location, as may be clearly observed. Again, we have the single (or collective) dipolar mode at 3.4 eV and the collective mode created by the cluster at 2.3 eV. The two curves match perfectly the optical results presented in Fig. 1.

## Conclusions

Individual and collective plasmonic modes of silver NPs aggregates were investigated at nanometer scale by means of EFTEM. Excellent agreement was found with UV–vis absorption optical measurements. The study covers from one single standing NP to a large cluster formed by more than 200 NPs. Both individual and collective modes were clearly identified allowing the identification of the main contributions to the collective mode. It was shown that the aspect ratio of the cluster and as its shape are the main contributors.

## Abbreviations

LSPR: localized surface-plasmon resonance
NP: nanoparticle
UV: ultraviolet
UV–vis: ultraviolet–visible
EELS: electron energy-loss spectroscopy
TEM: transmission electron microscopy
EFTEM: energy-filtered transmission electron microscopy
STEM: scanning transmission electron microscopy
SESAM: sub-electronvolt sub-Angstrom microscope
BF: bright field
